# Development of the International Cardiac Rehabilitation Registry Including Variable Selection and Definition Process

**DOI:** 10.5334/gh.1091

**Published:** 2022-01-11

**Authors:** Mohiul I. Chowdhury, Karam Turk-Adawi, Abraham Samuel Babu, Gabriela Lime de Melo Ghisi, Pamela Seron, Tee Joo Yeo, Jamal Uddin, Martin Heine, Marianna Garcia Saldivia, Evangelia Kouidi, Masoumeh Sadeghi, Raghdah Aljehani, Sherry L. Grace

**Affiliations:** 1 Faculty of Health, York University, Toronto, Ontario, Canada; 2 KITE-Toronto Rehabilitation Institute, University Health Network, University of Toronto, Toronto, Ontario, Canada; 3 QU Health, Qatar University, Al Jamiaa St, Doha, Qatar; 4 Department of Physiotherapy, Manipal College of Health Professions, Manipal Academy of Higher Education, Karnataka, India; 5 Faculty of Medicine, Universidad de La Frontera, Temuco, Chile; 6 Cardiac Department, National University Heart Centre Singapore, Singapore; 7 Department of Cardiac Surgery, Ibrahim Cardiac Hospital & Research Institute, Dhaka, Bangladesh; 8 Institute of Sport and Exercise Medicine, Faculty of Medicine and Health Sciences, Stellenbosch University, Stellenbosch, South Africa; 9 UMAE Hospital de Cardiología, Centro Médico Nacional Siglo XXI, IMSS, México; 10 Lab of Sports Medicine, Department of Physical Education and Sports Sciences, Aristotle University of Thessaloniki, Thessaloniki, Greece; 11 Cardiac Rehabilitation Research Center, Cardiovascular Research Institute, Isfahan University of Medical Sciences, Isfahan, Iran; 12 Cardiopulmonary Rehabilitation, King Abdullah Medical City, Mecca, Saudi Arabia

**Keywords:** Cardiac rehabilitation, registries, Delphi technique, patient-reported outcomes, Benchmarking, governance

## Abstract

**Introduction:**

The International Council of Cardiovascular Prevention and Rehabilitation (ICCPR) is developing a registry (ICRR) specifically for low-resource settings, where the burden of cardiovascular diseases is greatest and the need for program development highest. Herein we describe the development process, including the variable selection process.

**Method:**

Following a literature search on registry best practices, a stepwise model for ICRR development was identified. Then, based on recommendations by Core Outcome Set-STAndards for Development (COS-STAD), we underwent a process to identify variables. All available CR registries were contacted to request their data dictionaries, reviewed CR quality indicators and guideline recommendations, and searched for common data elements and core outcome sets; 35 unique variables (including patient-reported outcomes) were selected for potential inclusion. Twenty-one purposively-identified stakeholders and experts agreed to serve on a Delphi panel. Panelists rated the variables in an online survey, and suggested potential additional variables; A webcall was held to reach consensus on which to include/exclude. Next, panelists provided input to finalize each variable definition, and rated which associated indicators should be used for benchmarking in registry dashboards and a patient lay summary; a second consensus call was held. A 1-month public comment period ensued.

**Results:**

First, registry objectives and governance were approved by ICCPR, including data quality and access policies. The protocol was developed, for public posting. For variable selection, the overall mean rating was 6.1 ± 0.3/7; 12 were excluded, some of which were moved to a program survey, and others were revised. Two variables were added in an annual follow-up, resulting in 13 program and 16 patient-reported variables. Legal advice was sought to finalize ICRR agreements. Ethics approvals were obtained. Usability testing is now being initiated.

**Conclusion:**

It is hoped this will serve to harmonize CR assessment internationally and enable quality improvement in CR delivery in low-resource settings.

## Introduction

Cardiovascular diseases (CVD) incidence is increasing at an alarming rate in low and middle-income countries (LMICs) [1,2]. Correspondingly, years lived with disability due to CVD in LMICs has increased from 12.4 million in 1990 to 25.2 million in 2019 [3]. Cardiac rehabilitation (CR) is a proven model of care delivering secondary prevention to mitigate this burden [4]. Indeed, participation results in 20% lower mortality and morbidity [5], including re-hospitalization and revascularization, which are a major expense to health systems [6]. Evidence from LMICs also support these significant benefits [7].

The clinical benefits and cost-effectiveness of health services such as CR can be optimized where it is evidence-based, timely, patient-centered, equitable and otherwise of high-quality [8]. It is well-established that there is often a wide gap between clinical practice guideline recommendations and care, and this is also true for CVD secondary prevention [9], despite the relatively low cost of recommended therapies [10,11]. Registries serve as key means to understand and improve care [12]. Participating in registries has significant impacts on processes of care, and often on patient outcomes and care costs [13,14], including in the cardiac field [15,16,17,18,19,20,21,22].

Unfortunately, there are only eight CR registries globally, all national in scale [23], with only one in a LMIC (China). Therefore, based on member request, the International Council of Cardiovascular Prevention and Rehabilitation (ICCPR) recently embarked on a process to develop an International CR Registry (ICRR) for low-resource settings (i.e., LMICs or CR-deprived areas in high-income countries without existing CR registries) [24]. The objectives of this report are to describe the methods through which it is being developed, with regard to governance and the data dictionary.

## Methods

### Development and governance

First, a review of best practices in registry development and operation was undertaken. A search strategy for Medline was developed with direction of an information specialist; the search strategy is shown in Appendix 1. Policy-type papers were also considered, and therefore a grey literature search was also performed [25]. Results were used to inform development and governance processes.

#### ICRR variable selection and definition process

A rigorous, stepwise approach to ICRR variable selection and definition was undertaken, upon consideration of findings from the above review. Ethical approval was obtained from York University’s Office of Research Ethics (#e2020-147). We followed a process based on recommendations by Core Outcome Set-STAndards for Development (COS-STAD) [26], and considered the United States (US) Agency for Healthcare Research and Quality’s (AHRQ) Outcome Measures Framework (i.e., characteristics, treatment, and outcome domains) [12], which were identified through the above review.

First, we approached all 8 known existing CR registries, as identified in the review by Poffley et al. [23], to request their variable lists, to learn from their experiences with chosen variables and so some harmonization could be achieved where warranted; Upon contacting each, it was determined two were at the site (not patient) level, and that two registries were now defunct (Canada, Europe). Through a request to all 40 ICCPR member associations and 14 friends, some new registries were identified. Ultimately, all eight currently-active CR registries were contacted: United Kingdom (excluding Scotland), US, Austria, Sweden, Denmark, Australia, China and Japan (*https://globalcardiacrehab.com/Other-CR-Registries*). We informed them about ICCPR’s development of a registry, expressed our hope to collaborate going forward, and requested their variable lists confidentially. Responses received were tabulated exhaustively, such that assessment of each variable in each registry was computed; variables assessed in most registries were then listed in an initial potential variable list.

Next, as per best practices identified through the above review, to identify any key variables that may have been missed, we checked for common data elements recommended for all registries through the US National Library of Medicine repository (*https://cde.nlm.nih.gov/home*), and variable lists in core outcome sets for trials for CVD outpatients [27] through also the Core Outcomes Measures in Effectiveness Trials (COMET) initiative [28]. As per COS-STAD, we also considered the patient voice, by considering variables recommended in the International Consortium for Health Outcome Measurement’s standard set for coronary artery disease (*https://www.ichom.org/portfolio/coronary-artery-disease/*) and the peer-reviewed literature [29,30], as well as seeking input from patients in low-resource settings. Finally, we checked that we assess CR quality indicators assessed by CR associations globally identified in a previous review [31,32], and also assessed whether CR practice guideline recommendations were assessable in the initial potential ICRR variable list by cross-referencing the variable list with recommendations from the highest-quality CR guidelines relevant to low-resource settings [33], to be used by the World Health Organization (WHO) in their Package of Rehabilitation Interventions for ischemic heart disease [34].

Then, as per COS-STAD recommendations [26], a 2-stage consensus process was undertaken to finalize the variable list [35], associated definitions/measurement tools, and to select indicators from the list to be reported in ICRR reporting dashboards for participating CR programs. In accordance with best practices [35], through ICCPR council, a panel of CR stakeholders (e.g., policy-makers, CR managers), multi-disciplinary CR providers (working at a CR program meeting ICRR site inclusion/exclusion criteria preferably; i.e., knowledge users) and experts (e.g., researchers, guideline developers) was assembled purposively, with a goal to have 20 panelists from primarily low-resource settings across all WHO regions, and/or with expertise in other CR registries [23] or CR quality indicators (corresponding authors of those invited) [32]. Panelists were informed how the two steps would be undertaken before the consensus process was initiated, and had the opportunity to provide input. The rating process was done in accordance with best practices by the Canadian Cardiovascular Society [36].

At the first stage, an online survey was emailed to all panelists in June 2020 through REDCap [37], prefaced by an online consent form. Agreeing panelists were then requested to rate each potential variable on a scale from 1–7 in terms of feasibility of collecting, importance/relevance to CR delivery quality, actionability (i.e., if the variable were included, could the program use the resulting information to improve care), and evidentiary basis for including the variable, as well as an overall rating. Panelists were also asked to provide any open-ended comments on the variables and to specify any variables they perceive ICRR should consider adding.

Ratings were then collated; we planned to consider variables with overall average scores ≥5/7 to have consensus for inclusion, with those <4 as having consensus for exclusion, however both were to be confirmed via videocall, to consider the overall set of variables [36]. All other variables would be considered to have ‘unclear consensus,’ and with consideration of free-form comments and other ratings which were circulated anonymously, were to be discussed on the first videocall until consensus for inclusion or exclusion was achieved. The first videocall was held in July 2020, after circulating anonymous rating results (means and standard deviations), as well as a collated list of additional variables suggested by panelists. The senior author (SLG) chaired the call, and aimed to facilitate fulsome deliberation with all perspectives voiced, to achieve consensus on the final list of included variables.

At the second stage, a summary of decisions from the first stage was circulated, along with a proposed definition of each included variable, developed based on definitions in publicly-available CR registry data dictionaries, data element repositories listed above where variables matched, or brief psychometrically-validated scales where possible [38]. A list of proposed indicators based on the variables selected and existing CR quality indicators was also circulated for potential inclusion in the ICRR reporting dashboards [31,32], and a post-program lay summary drafted to summarize patient progress and areas for continued risk reduction based on outcomes of importance to patients (see: *https://globalcardiacrehab.com/ICRR-for-Patients*) for panelist input [29,30]. They were also asked to vote whether chosen dashboard indicators should be reported where applicable as: (a) percentage of patients at target post-program, (b) mean change score from pre to post-program, or (c) change in percent of patients at target from pre to post-program. Responses received were integrated into the data dictionary; this and indicator suggestions were circulated to panelists to discuss before the second and final consensus call which was held in September 2020.

Finally, with all input integrated and agreed by the panelists, a 1-month public comment period was held. The ICRR data dictionary was posted to the ICCPR website with an invitation to provide input. Panelists, ICCPR council members, and CR registry contacts were alerted via email and social media to share the call widely. CR patients from low-resource settings with English-language proficiency were invited to give input on the patient-reported items for each assessment point, as well as the lay summary.

## Results

### Best practices in registry development and governance

**Figure 1:**
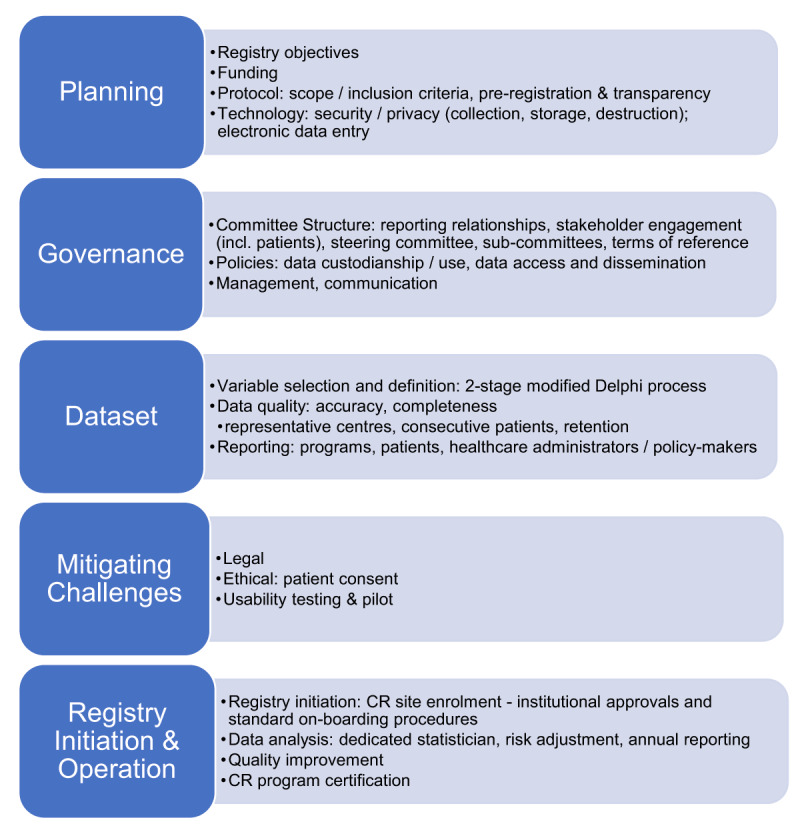
Five-Step Registry Development Process with ICRR Activities. **Legend:** * Based on Mandavia et al. [43] and AHRQ’s 4^th^ edition Registry User’s Guide{Formatting Citation}. CR = cardiac rehabilitation, ICRR = International Cardiac Rehabilitation Registry.

Following the literature search, seven key documents were identified for particular consideration in ICRR development and operation [12,39,40,41,42,43,44]. Based on those, and in particular the US AHRQ [12] Registry User’s Guide and Mandavia et al. [43] which was developed through an evidence-based process, a 5-step development process was finalized (***Figure 1***); some activities were undertaken concurrently.

### Planning and governance

First, registry objectives were explicated; input was solicited from ICCPR Executive, who ultimately approved them. They can be found in the protocol on the ICRR website at: *https://globalcardiacrehab.com/ICRR-Governance*.

**Figure 2:**
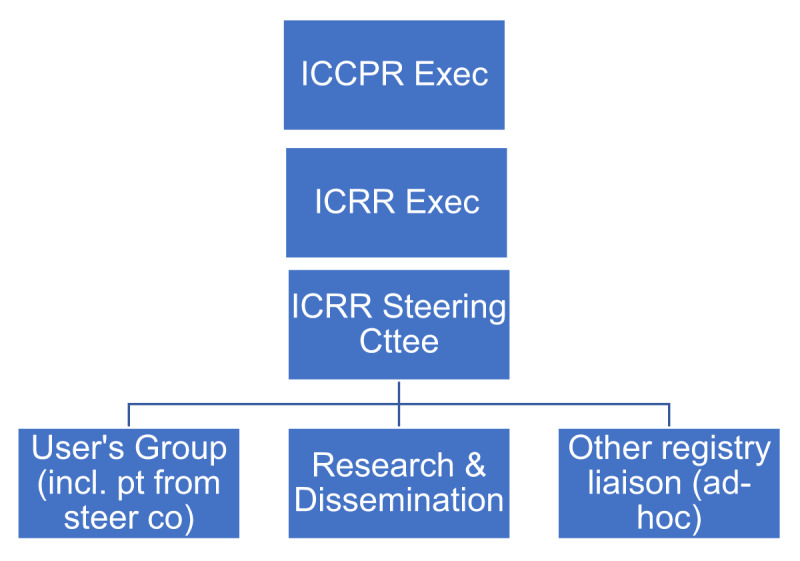
ICRR Governance organization chart. **Legend:** ICCPR = International Council of Cardiovascular Prevention and Rehabilitation, ICRR = International Cardiac Rehabilitation Registry. Incl., including; pt, patient, co., committee, exec., executive.

Next, ICRR governance was developed (***Figure 2***). ICRR will report in, and be accountable to, the ICCPR Executive committee. The ICCPR Executive deliberated and confirmed ICRR co-chairs (SLG, who is on ICCPR Executive since inception and formerly served with Canada’s CR registry, and KTA, a friend of ICCPR who does research with a CR program in a low-resource setting), who had developed a grant proposal to secure funding to develop and pilot the ICRR, which had been considered by Executive prior to submission (***Figure 1***). The ICRR co-chairs developed the terms of reference for the ICRR Steering Committee, which were approved by ICCPR Executive (see: *https://globalcardiacrehab.com/ICRR-Governance*). They proposed three sub-committees, one for users (i.e., on-boarding and quality improvement), one for research (e.g., data quality and access), and a final ad-hoc liaison sub-committee to support collaboration and communication with other CR registries internationally (as per initial communications outlined above). This structure was shared with ICCPR Council, and approved by ICCPR Executive.

The co-chairs then contacted several registry software vendors to secure a host for ICRR; upon review of privacy and security protections as well as demonstration of platforms, electronic data upload capability and reporting features, Dendrite Clinical Systems was selected (*https://www.e-dendrite.com/*), with approval from ICCPR Executive. The contract for development included two reporting dashboards (six indicators each; i.e., patient-related and program-related outcomes), with two comparisons for each indicator, as well as variable completeness reports at the site-level.

Next, the co-chairs began drafting parts of the ICRR protocol to inform the variable selection process, outlining the definition of low-resource setting,’ [24] as well as CR program and patient inclusion and exclusion criteria; Ethical issues, proposed procedures, privacy and security assurance processes, as well as reporting goals were also preliminarily delineated (see: *https://globalcardiacrehab.com/ICRR-Governance*). This was shared with Delphi panelists.

### Dataset: Variable selection and data definitions

Variable lists were received from six of the eight currently-existing registries (including China). Compilation resulted in a list of 66 unique variables. ICCPR Executive deemed this to be too long a list for a low-resource setting, and therefore requested the ICRR co-chairs reduce this list; upon consideration of the most-frequently assessed variables (11 were assessed across all six, and seven were considered in at least five of the six) as well as variables commonly assessed in the other sources outlined above, this culminated in a list of 35 variables (***Table 1***).

**Table 1:** Initial variable list with overall ratings and inclusion decisions.


VARIABLE NUMBER IN DATA DICTIONARY (PROGRAM SURVEY)	VARIABLE NAME (ASSESSMENT POINT)	OVERALL RATING MEAN ± STANDARD DEVIATION	INCLUSION DECISION

1	Year of birth ǁ (pre)	6.7 ± 0.7	I

2	Sex ǁ (pre)	6.8 ± 0.4	I

4	Referral diagnoses (pre)	6.7 ± 0.6	I

5	Referral intervention(s) (pre)	6.5 ± 0.8	I

	Referral date*	6.0 ± 1.0	E

3	Initial assessment date (pre)	6.2 ± 0.9	I

7, 27, 29	Interim event(s)/procedures/vital status* (post)	6.0 ± 0.8	R

7	Premature program termination/Program Completion‡ (post)	6.4 ± 0.9	I

(28, 29)	Number of prescribed exercise sessions	6.2 ± 1.0	P

6	Number of completed exercise sessions‡ (post)	6.4 ± 0.8	I

(25)	Resistance training	6.2 ± 0.9	P

(25)	Education provided	6.4 ± 0.8	P

(25)	Stress management provided	6.1 ± 0.9	P

(25) 23	Cardiac medications†‡^˄^	6.7 ± 0.7	P, R

8	Low-density lipoprotein^˄^ (pre, post, annual)	6.2 ± 1.2	I

9	Waist circumference*‡^˄^ (pre, post, annual)	5.9 ± 1.1	R

10	Systolic blood pressure‡^˄^ (pre, post, annual)	6.4 ± 0.7	I

10	Diastolic blood pressure^˄^ (pre, post, annual)	6.4 ± 0.7	I

11	Peak METs‡ (pre, post)	6.1 ± 1.0	I

13	Marital status* (pre)	5.6 ± 1.3	R†

14	Educational attainment* (pre)	6.0 ± 1.1	I†

24	Work status (pre, post, annual)	6.1 ± 0.9	I†

15	Comorbidities (pre)	6.4 ± 0.6	I†

16	Economic security* (pre)	5.6 ± 1.1	R†

17	Medication coverage* (pre)	5.8 ± 1.3	R†

–	Health literacy*	5.9 ± 1.1	E

18	Quality of life‡^˄^ (pre, post, annual)	6.1 ± 0.8	I†

19	Depressive symptoms^˄^ (pre, post, annual)	6.3 ± 0.7	I†

20	Fruit and vegetable consumption*‡^˄^ (pre, post, annual)	6.0 ± 0.8	I†

–	Processed food consumption*	5.3 ± 1.0	E

21	Physical activity‡^˄^ (pre, post, annual)	6.1 ± 1.3	I†

22	Tobacco use‡ (pre, post, annual)	6.8 ± 0.5	I†

25	Cardiac knowledge‡^˄^ (post)	6.1 ± 1.0	I†

–	Program satisfaction*	5.9 ± 0.9	E

26	Cardiac rehabilitation model (post)	6.1 ± 1.0	I†


I, included; E, excluded, R, revised, P, moved to program survey (one time). * Discussed during consensus call. † Patient report. ǁ Mandatory variables. ‡ Included in ICRR dashboards. ^˄^ Included in patient lay summary.

Twenty-eight panelists were invited to serve, of which 21 served in both rounds, representing stakeholders and regions as targeted. Overall ratings from round one are shown in ***Table 1***. As shown, most variables were rated above five, and therefore it was decided by ICRR Executive to change the threshold for variable inclusion to six, and this was approved by panelists. Additional variables suggested included ethnocultural background, barrier assessment, risk assessment/stratification, vaccination, sleep, blood glucose, alcohol consumption, anxiety, and support.

During the first stage videocall, panelists first discussed the optimal number of variables for low-resource settings, as raised by ICCPR Executive. Panelists distinguished number by source: they suggested 7–11 by programs (not including baseline characteristics), and recommended variables be directly reported by patients where feasible, noting literacy and language barriers will have to be carefully considered given the context(s); patient report would be optional to sites and patients, with sites supported to collect patient data via interview or survey where more feasible for institutional, ethical or patient-related reasons. It was suggested that structural indicators be assessed once in a program survey to be completed at the time of CR program on-boarding to the ICRR.

Variables discussed are shown in ***Table 1***. Of the 20 program-reported variables in the initial list, nine were excluded, of which five were moved to the program survey (see Table; available here: *https://globalcardiacrehab.com/ICRR-Variables-&-Data-Dictionary*). Of 16 potential patient-reported variables, three were excluded. Finally, one program-reported variable (i.e., waist circumference changed to body mass index) and one patient-reported variable (marital status revised to social support) were revised. Additional variables agreed were cardiac symptoms and morbidity (both patient-report where possible, annual); these outcomes are important to patients [29,30]. This resulted in a list of 13 program-reported variables, and 16 patient-reported variables agreed by panelists. Assessment points are shown in ***Table 1***, in accordance with AHRQ’s framework (i.e., characteristics at pre-program assessment, treatment at post-program, and outcomes at each assessment point) [12].

**Figure 3:**
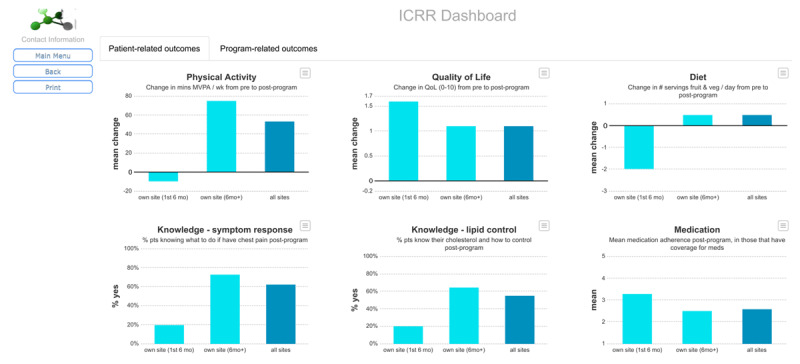
ICRR Patient-Related Indicator Reporting Dashboard. **Legend:** ICRR = International Cardiac Rehabilitation Registry. *Note*: Dummy data are shown.

During the second-stage videocall, first assessment points were discussed. Long-term data is desirable, but feasibility of collection by programs in low-resources settings may be limited. Panelists agreed that the annual assessment move forward -- in addition to the pre and post (or ‘progress’ if patient did not complete the program)-program assessments-- comprising only one program-reported variable (health status), two additional patient-reported variables (symptoms and morbidity) and repeat of other patient-reported outcome variables where patients are willing. They defended their decision based on the fact that variables assessed at that point are optional and with the understanding retention bias will need to be monitored. Minor edits to variable definitions proposed by panelists were approved (e.g., broadening some terminology to be applicable across more contexts). Indicators for both dashboards were also approved, and the decision that they report mean change from pre- to post-program was ratified (***Table 2***). Comparisons to site data were also finalized as: (1) to all other programs and (2) to own program over time (first six months of data entered, compared to all subsequent data, to capture any quality improvement; ***Figure 3***). The patient lay summary was also agreed, with some revision (e.g., addition of site-specific logos and contact information).

**Table 2:** Indicators selected for ICRR dashboards based on variables chosen by Delphi panelists.


	PROGRAM-RELATED	VARIABLE #†	PATIENT-RELATED§	VARIABLE #†

*1*	Proportion of patients completing the program (i.e., attended post-program assessment) [45].	7	Change in minutes of moderate or vigorous-intensity physical activity per week from pre- to post-program.	21

*2*	Change in peak METs from pre- to post-program.	11	Change in number of servings of fruits and vegetables per day from pre- to post-program.	20

*3*	Change in systolic blood pressure from pre- to post-program.	10	Change in mean quality of life score from pre- to post-program.	18

*4*	Change in body mass index from pre- to post-program.	9	Proportion of patients knowing what to do if they have chest pain post-program.	25

*5*	Change in % of patients reporting current tobacco use from pre- to post-program.	22	Proportion of patients knowing how to control their lipids post-program.	25

*6*	Number of supervised CR sessions attended*.	6	Mean medication adherence (1–5) post-program.	23


ICRR = International Cardiac Rehabilitation Registry. * Not compared to other sites, as number prescribed varies by program; only compared to own program first 6 months in ICRR versus after 6 months. † As per data dictionary found in Appendix 2. § Shown in Figure 3. *Note*: Exclusions not shown, but are available from the corresponding author.

The list of variables and definitions was then posted for public comment, and the patient surveys and lay summary shared with patients meeting inclusion/exclusion criteria as outlined in the protocol; feedback was integrated by ICRR Executive. The final list of variables and their definitions, along with source and assessment points are shown in Appendix 2 (and posted publicly at *https://globalcardiacrehab.com/ICRR-Variables-&-Data-Dictionary*; any future potential revisions following usability testing will be posted there).

### Bringing it all together and mitigating challenges

As per ***Figure 1***, all activities for the planning, governance and dataset steps were then completed. For instance, a call was put out for interested ICCPR Council members and friends as well as Delphi panelists from low-resource settings to self-nominate for the ICRR Steering committee. The ICRR Executive vetted all received nominations, considering needed representation on the committee based on the approved Terms and declarations of any conflicts of interest. They forwarded a slate of potential members including sub-committee chairs, for ultimate approval by ICCPR Executive; inaugural members are shown here: *https://globalcardiacrehab.com/ICRR-Governance* (note ICCPR Treasurer is supporting ICRR).

Next, ICRR Executive worked with the sub-committee chairs to develop terms for those committees (see: *https://globalcardiacrehab.com/ICRR-Governance*), and processes to constitute the initial sub-committees. Patients were engaged on the user sub-committee. The research sub-committee terms include engaging a statistician for rigorous, risk-adjusted analyses and annual report generation (through which high-performing programs shall be recognized). The research sub-committee chair developed a policy on data access and dissemination as well as data quality, which were approved by ICRR Steering and ICCPR Executive committees (see: *https://globalcardiacrehab.com/ICRR-Governance*).

ICRR Executive worked with the user sub-committee to develop the ICRR website on the ICCPR website (patients gave input on patient page), as well as start a social media presence (e.g., Twitter #ICRregistry). They also drafted information for CR sites interested in joining ICRR for posting on the website (see: *https://globalcardiacrehab.com/ICRR_sites*), as well as a standard operating procedure for on-boarding, which will be pilot-tested as outlined below. This includes procedures to ensure sites enroll consecutive patients, enter data consistently with the data dictionary, and provide as complete follow-up data as possible annually. They also developed an ICRR – CR program/site agreement, to be approved by CR program institutions prior to on-boarding, covering ethical requirements, data custodianship, and security, among other matters; a lawyer was consulted to finalize this, as well as an agreement between ICRR and Dendrite regarding data processing (e.g., secure data storage, eventual data destruction, handling patient requests for data- see: *https://globalcardiacrehab.com/ICRR-for-Patients*).

The protocol was then finalized based on the above decisions; The measures were added, and assessment points. It was then circulated to ICCPR Executive and the ICRR Steering Committee; the protocol was discussed at a meeting of both committees, and ultimately approved following integration of feedback (found at: *https://globalcardiacrehab.com/ICRR-Governance*). The protocol was submitted for research ethics approval in ICCPR’s home country, Canada (York University Office of Research Ethics approval number e2020-359; ***Figure 1***). The protocol is flexible, in that each ICRR-participating site will have to follow their locally-approved procedures with regard to patient opt-out versus written patient consent where the site is using patient-report; an information letter and consent form have been approved centrally, that can be then adapted for each site (both available at *https://globalcardiacrehab.com/ICRR-for-Patients*). The protocol was then registered prospectively with *clinicaltrials.gov* (NCT04676100).

ICRR Executive has worked with Dendrite to develop the ICRR (data definitions embedded), including patient report (email and reminder email text with link to the surveys circulated to patients on user sub-committee for input), the indicator reporting dashboards (***Figure 3***), and the patient lay summary (some of their survey responses and change scores are pre-populated). Each patient is given a unique registry identification number, given names or other identifying information are not collected for privacy reasons. Out-of-range limits are applied for all continuous variables; all other variables are forced choice to minimize data entry errors (no free-text fields). Provision is made so when new sites are on-boarded, Dendrite will ensure the post-program assessment is triggered based on the number of weeks duration of the given program, as reported in the program survey; provision is also made so that patient-reported surveys are not sent to any patients who have not consented (and also could not be if the site does not have ethics approval for this). Sites see a listing of their patient entries upon login with their unique credentials, which is colour-coded to denote whether each assessment point is complete and when the next assessment is due for each patient. The data completeness reports Dendrite created will also be made available to the research sub-committee for auditing as per the data quality policy.

## Discussion

The ICRR has been developed following a rigorous, evidence-informed process. We are now poised for usability testing, as per the fifth and final step in the ICRR development process (***Figure 1***). This will be achieved using a ‘think-aloud method,’ [46,47,48] where CR staff will anonymously enter a patient’s data into the ICRR demonstration site on a videoconferencing platform, and then undergo a semi-structured interview. Interview audio-recordings will be transcribed, and analyzed using content analysis concurrently with collection. Results will be used to refine the registry, with interviews continuing until no further issues that can be addressed are raised.

### Future directions: Registry initiation and operation

Once feedback and findings from the usability tests are integrated into the ICRR, we will have a ‘soft launch’ of sites known to ICCPR. ICRR will be open to initiation by any site once any site initiation challenges are worked out. The registry will be marketed through members of ICCPR’s >40 member associations and >15 friends, as well as their CR email distribution list reaching <1500 programs around the world. It will also be shared with the CR community though an ICCPR webinar, and the recording posted to our website.

Once a reasonable amount of data has been accumulated, the user sub-committee co-chair will peruse the dashboards to get a sense where quality is lowest, and for which of these indicators there are known strategies to mitigate the quality issues. The co-chair shall confer with the user sub-committee to develop a quality improvement plan for approval, including a webinar. Thereafter, the co-chair shall invite interested sites (including those who may not be performing as well as others) to a ‘learning community’ to work on changing processes of care to address the quality issues (*https://globalcardiacrehab.com/resources/Documents/ICRR_QI%20plan_v1-2.pdf*). This will be an on-going process twice per year.

In addition to availability of the patient discharge lay summary, outcome benchmarking dashboards, and quality improvement supports, other incentives may be needed to stimulate adoption of the registry. Programs also have access to their own data at all times easily exportable in MS Excel, and are invited to be involved in international research. Moreover, ICCPR has developed a program certification process for interested sites using registry data (from the program survey and patient-level data) in addition to a virtual site visit (*https://globalcardiacrehab.com/Program-Certification*). Charges, on a sliding scale, are commensurate with the cost to run the ICRR, and re-certification would be required every three years. Previously, CR program certification was only offered by the US; While non-US programs can apply, the requirements and certification costs (i.e., USD$935 for initial certification and USD$835 for regular re-certification) may be prohibitive in low-resource settings, and the standards less relevant.

### Limitations

The ICRR will have some limitations. First, generalizability may be a challenge. Site participation will be voluntary, and therefore they may not be representative of the average program in low-resource settings or in different regions of the globe [49]. However, there is some evidence suggesting site selection in registries may not lead to considerable bias [50,51]. Moreover, we know there are biases in patient access to CR [52], and hence also patients in the registry may not be representative of the average cardiac patient in these settings. Second, target CR programs may have difficulty participating due the fact that they have few resources, and due to known barriers to CR registry uptake [53,54]. ICRR will be available to sites at no cost, and interested sites will be thoroughly informed about the resources needed to participate and supported in participating prior to on-boarding. ICRR has attempted to build in flexibility to meet the needs of programs in low-resource settings, and ensure sites reap benefits of participation that counter the time required (e.g., data on their program efficacy, including in relation to other programs internationally, taking part in quality improvement activities, availability of lay summary for their patients). Third, due to the international scope of the registry and limited resources, ICRR will be unable to audit whether sites are entering all patients consecutively, or the accuracy of entered data. To mitigate this, training procedures are thorough, and a data quality policy has been developed for ongoing quality assurance. We attempted to ensure the data dictionary and instructions are very clear and leave little room for error. The registry itself constrains responses to reduce the chance of error in data entry.

Fourth, at this point the patient-reported variables are only available in English. Many cardiac patients in low-resource settings lack English-language proficiency. ICRR hopes to secure funds and work with our partners to translate the patient-reported items and ensure the translations are of high-quality. In the interim, if approved by their institution, sites can administer the patient-reported items via interview. Fifth, it is likely that retention will be a challenge in low-resource settings. Without good follow-up data, the utility of the ICRR will be lessened. To mitigate this, for patients approved and consenting to provide data, the ICRR does send an email and/or text at all assessment points regardless of program completion, until a patient is denoted as having died or being too ill to complete further assessments; a reminder is sent to non-responders. Moreover, strategies to collect follow-up variables via phone where patients do not come in are suggested in the data dictionary.

## Conclusion

Through collaboration and following this multi-step process based on best practices, an internationally-agreed minimum set of variables for CR program evaluation has been established for the first time. The ICRR governance structure has been developed, and is made transparent to the global CR community. It is hoped the ICRR data dictionary will enable harmonization of CR assessment internationally, and that the registry will enable quality improvement in CR delivery.

## Additional Files

The additional files for this article can be found as follows:

10.5334/gh.1091.s1 Appendix 1. Search Strategy.

10.5334/gh.1091.s2 Appendix 2. Data Dictionary.
