# Supplementary material for: Development of the International Cardiac Rehabilitation Registry Including Variable Selection and Definition Process
Source: Glob Heart. 2022 Jan 11;17(1):1. doi: 10.5334/gh.1091 (PMC8757385; doi:10.5334/gh.1091)
Supplement: Appendix 1. — Search Strategy. [file gh-17-1-1091-s1.pdf]

Appendix 1: Registries & Effect in CVD Populations/outpatients Search Strategy: Ovid MEDLINE(R) ALL <1946 to October 16, 2020>

----

1 exp *Cardiovascular Diseases/ (2049229)
2 ((disorder* or disease*) adj3 (cardiovascular or heart or coronary artery or cardiac or cerebrovascular)).ti,kw. (145966)
3 ((heart or cardiac or myocardial) adj3 failure).ti,kw. (74367)
4 (ischemi* adj3 (disease* or heart or myocardial)).ti,kw. (25867)
5 (coronary adj3 (arterioscleros* or atheroscleros*)).ti,kw. (4205)
6 stroke.ti,kw. (113547)
7 (cerebrovascular adj3 (accident* or apoplex*)).ti,kw. (1383)
8 or/1-7 (2108243)
9 Registries/ (91150)
10 ((registry or registries) adj4 (treatment or stroke or clinical)).tw,kw. (18326)
11 9 or 10 (105558)
12 *"Quality of Health Care"/ (37796)
13 *Quality Assurance, Health Care/ (32191)
14 quality-improvement.ti,kw. (15327)
15 (quality adj3 (improv* or care or assur* or healthcare or indicator* or monitor* or impact*)).ti,kw. (50693)
16 (improv* adj3 (care or performance)).ti,kw. (17931)
17 or/12-16 (120026)
18 Outpatients/ (16286)
19 exp treatment outcome/ (1068782)
20 (outpatient* or out-patient* or "out patient*").tw,kw. (186912)
21 ((clinical or treatment) adj3 (effect* or efficac* or outcome*)).tw,kw. (698413)
22 or/18-21 (1751484)
23 8 and 11 and 17 and 22 (181)

****
