# Supplementary material for: Development of the International Cardiac Rehabilitation Registry Including Variable Selection and Definition Process
Source: Glob Heart. 2022 Jan 11;17(1):1. doi: 10.5334/gh.1091 (PMC8757385; doi:10.5334/gh.1091)
Supplement: Appendix 2. — Data Dictionary. [file gh-17-1-1091-s2.pdf]

International Council of Cardiovascular Prevention and Rehabilitation (ICCPR)

International CR Registry (ICRR)

VARIABLES & D E F I N I T I O N S 8.0

Including data entry instructions

July 15, 2021
(post-registry build; peri-usability testing and pre-pilot)

ICCPR Intellectual P

Copyright © International Council of Cardiovascular Prevention and Rehabilitation
To be used with permission only. Not for re-distribution

INSTRUCTIONS / INFORMATION BEFORE YOU BEGIN

For any questions about the registry variables contact iccpr.icrr@gmail.com; for questions on access to the registry / logins, contact national-support@e-dendrite.com. For instructions on how to use other registry features, see the "ancillary features" file.

There is a powerpoint file that has a software training manual on how to navigate the screens (but this is described to follow here too). From the main menu, select "enter clinical data" (blue rectangle) to get started. You will see your roster of patients there on your "my patients" screen, or can "add new patient" (top left, green rectangle). (Please note if you wish to delete a patient, we will need to contact Dendrite to do so [this is to prevent accidental deletion of data]); data stewards can only see patients from their site.

We have purchased 5 licenses, and therefore 5 users across all sites can be in the registry at any one time. If you can't get on (you will see a message informing you all licenses are engaged), check back a bit later. We are hoping this is sufficient given this is international, and hence sites will be in different time zones. If you often cannot get in, please email us so we can consider how to secure funding to purchase another license.

Please note that as per the protocol, all referred patients meeting inclusion / exclusion criteria (e.g., phase II cardiac patients) should be entered in ICRR if possible, so that we can assess the proportion of referred patients enrolling (CR quality indicator). For those patients who do not enroll, please complete all available information from their referral form. It is hoped that gender, year of birth, referral diagnosis / procedure could be completed.

Only the gender and year of birth variables are mandatory, but of course we encourage as complete data provision as possible to optimize the utility of the ICRR. If you do not have the information for a variable, leave it blank (there are only a couple of variables where you can specify "none of the above" for example); do try to secure the information if possible and go back in later to enter it. See the dashboard document for information on how to see data completeness.

All definitions are shown below in black, with instructions in green. Many variables have a blue "i" in a white bubble; hover over it to see the definitions from this document on screen.

For all variables you select a response option or enter a number (you cannot enter ranges as that would render data analysis and dashboard reporting problematic); there are only 3 open-ended text fields (variable 12 is an optional variable sites can use as they wish if desired at pre and post/progress, and variable 15 to specify "other" comorbidities). All continuous values have minimum and maximum values to prevent data entry errors.

Variables are all available in the registry on specific pages, by assessment point (pre-program, post/progress or annual). We have specified below in the table of contents on which registry page you can find the variables, and it is also shown in orange font below. As you are entering you can click "next" to get to the next page. When you are in a record for a patient, you can also skip to any page at any time, using the white rectangle drop-down menu in the top middle of any registry page. At any time you can click "save & exit" (blue rectangle, bottom right) and any entered data will be available under that patient ID on the "my patients" screen; you can go back in any time to enter more data.

Timing of assessment of the below variables is shown in yellow highlight in the ensuing data dictionary, and time windows are stated. There is also information regarding when / how assessments stop if patients are unfortunately too ill or die.

Source of information is shown in blue font (program, and potentially patient). For patient-reported variables, follow what you have approved with your local ethics board (e.g., email them the surveys). If patients do not have proficiency in English, you may ask the questions of the patient directly

Copyright © International Council of Cardiovascular Prevention and Rehabilitation
To be used with permission only. Not for re-distribution

ICCPR Intellectual Property

in-person, on the phone or video call if that is approved at your institution, and enter responses directly into the registry. If patients have English-language proficiency but do not have technology skills or access, ICRR can provide you paper versions of the items at each assessment point for their completion, and then someone from your program with an approved ICRR login can enter the data. Just ask!

Note you can go in at any time to edit data you have entered if you made an error.

Table of Contents:

a. *Program*-reported variables *pre*-program start on p. 4

b. *Program*-reported variables *post*-program (or denote reason they dropped out) start on p. 7

c. *Program*-reported *clinical* outcome variables assessed *pre and post*-program/progress start on p. 9

d. Patient-reported (or program entry if not possible) variables *pre*-program start on p.11

e. Patient-reported clinical variables *pre and post/progress* (even if they didn't finish)-program start on p.13

f. Other patient-reported (or program entry if not possible) variables *post-program* start on p.15

g. *Program* and patient-reported (or program entry if not possible) variables assessed *annually* start on p. 16 and 17, respectively

In the registry, program-reported variables pre-program appear on pages 1, 2. Variables on which patients can report pre-program are found on pages 3,4. Program-reported variables post-program/progress appear on pages 5,6. Variables on which patients can report post-program/progress are found on pages 7,8. Page 9 has the program report variable for the annual assessment. Pages 10,11 has the variables for the annual assessment for which patients can report.

ICCPR Intellectual Property

Copyright © International Council of Cardiovascular Prevention and Rehabilitation
To be used with permission only. Not for re-distribution

PROGRAM-REPORTED VARIABLES

PRE-PROGRAM/INTAKE ONLY

From the main menu, select “enter clinical data” (blue rectangle) to get started. You select “add new patient” (top left, green rectangle). These first 2 variables will appear

1. **Year of Birth**
   Select the patient’s year of birth (or best estimate if patient does not know and it is not recorded). This variable is mandatory
   Data Entry: 4 digits

2. **Gender**
   Select the patient’s sex. This variable is mandatory.
   Data Entry: Choices available are (choose 1):
   - ☐ Male
   - ☐ Female
   - ☐ Other /unknown

Click “enter registry data”.
The below variables are all found on page 1 of the registry.

3. **Initial assessment date**

   Enter the date the patient had their initial visit with the CR program for assessment of risk, history, etc. This could be in-person or remote.

   Note: this date will be used to determine when to trigger follow-up assessments of patients, based on duration of your program as denoted in the program survey from this date.
   Note 2: If no initial assessment do not enter a date. Even if patients do not enrol, if they are open to completion of patient-reported surveys for comparative purposes and this is approved through your local ethics board, please denote that by providing their contact information in the designated spot.

   Data Entry: Enter date using the calendar picker. The double “<<” on the outside at the top are for selecting year, and the inner single “>” and “<” are to move forward or backward by month.

4. **Referral Diagnoses - Cardiac**
   The referral diagnosis refers to the most recent <u>cardiac</u> diagnosis preceding the patient’s referral to cardiac rehabilitation. There may be more than one possible referral diagnosis reported if the second occurred within the same hospitalization period. DO NOT report historical diagnoses. DO NOT enter high-risk/ primary prevention patients, as they do not meet inclusion criteria (see protocol for further details). Non-cardiac comorbidities are reported in variable 15.

   Data Entry: Choices available are (select all that apply):
   - ☐ Stable coronary artery disease or stable angina
   - ☐ Acute coronary syndrome (ACS; e.g., myocardial infarction)

ICCPR Intellectual Property

Copyright © International Council of Cardiovascular Prevention and Rehabilitation
To be used with permission only. Not for re-distribution

☐ Heart failure
☐ Other (e.g., arrhythmia, valvular disease)

5. **Referral Intervention(s)**

Report cardiac interventions or procedures preceding the patient's referral to cardiac rehabilitation (no longer than 1 month). There may be more than one possible referral procedure reported if the second occurred within the same hospitalization period (check all that apply). DO NOT report interventions in the past, as they cannot be reliably captured in all patients.

Data Entry: check all that apply

- Percutaneous coronary intervention (PCI)
- Bypass surgery (CABG)
- Valve surgery or intervention
- Heart transplant
- Mechanical circulatory support (e.g., VAD)
- Rhythm Device insertion (e.g. CRT, ICD, pacemaker)
- Ablation
- Other
- None

0. **Patient data source**

☐ Patient (if this is selected, you will be prompted to provide mobile # and/or email address)
☐ Program is entering patient-reported data (ie., you will ask the questions of the patient)
☐ Neither (i.e., no patient-reported data will be provided)

IF NEITHER IS SELECTED
The patient-related pages of the registry will not be available to complete.

If this was an error and you wish to enter patient data, change your selection. Then you have to click either "Next Page" or "Save & Exit" before the drop-down list at the top of the page is re-populated with the patient-related pages.

IF PATIENT IS SELECTED:
Please note that texts cost the ICRR, so we prefer email, and the registry is set up to contact patients via email first. If there is no response after a week or 2, a text will be sent.

When entering a mobile number, it should be in this format: + CCC NNNNNNN, as follows:
It is necessary to prefix it with a "+" denoting it's an international number.
"CCC" is the international dialling code for the country (as many digits as required).
"NNNNNNN" is the number including any area code. We haven't constrained the number of digits, as the number template differs from country to country.
There should be no prefixed "0" or "1".

ICCPR Intellectual Property

Copyright © International Council of Cardiovascular Prevention and Rehabilitation
To be used with permission only. Not for re-distribution

Once patient contact information is entered, the patient-reported survey for pre-program will be sent by the registry to the patient by the next day. It will come from: icrr@e-dendrite.com on behalf of DCS Intellect Web <noreply@e-dendrite.com>. The subject line will read “Cardiac Rehab Registry”. Perhaps inform your patient to watch for it.

This will no longer be available 14 days from initial assessment date as it is assumed health behaviour would have changed from baseline. If no initial assessment date is entered, the registry will work from the first date you entered any data on the patient.

Note you can see a log of when correspondence is sent to patients, and if they respond, including dates, by clicking the blue rectangle named “pt report log” from the main menu (see file about registry screens for further information).

Also note that if the patient is providing data, the post-program survey will not be sent to patients if you specify “death” as cause of premature program termination on page 5 of the registry (program conclusion) for variable 7. The annual surveys will not be sent if you specify “patient died” or “patient to ill to complete further assessments” on any annual follow-up (page 9 or the registry, variable 27). This information is also outlined with the variables below for clarity.

Patients may also request to stop contributing data at any time and/or to withdraw previous data. ICRR has a process in place to enact this, which involves removing any email or text # from this variable. Patients can request this at any time at: https://globalcardiacrehab.com/ICRR-for-Patients. We will be in touch as needed if we receive any such requests, and request you contact us if you receive such requests.

If the patient is alive and has not requested to stop receiving surveys to you or to ICRR between now and the end of when their program would be done (according to the timing provided for your program duration), they will receive the post-program survey at the above contact information. See below for more information.

ICCPR Intellectual Property

Copyright © International Council of Cardiovascular Prevention and Rehabilitation
To be used with permission only. Not for re-distribution

PROGRAM-REPORTED VARIABLES
POST-PROGRAM ONLY (PROGRESS)

You informed ICRR of the number of weeks duration of your program in the initial program survey. Your site is set up so the post-program assessment will be due the specified number of weeks from the initial assessment date (if no initial assessment date is provided, it will be based on the date you first entered in their year of birth and gender). On the patient search / "my patients" listing page, the "post-program assessment status" column will show as "assessment due" when it is the time to complete this assessment; there is no active alert to you to go in and enter that, so if you are worried about forgetting you can put a reminder in your electronic calendar (patients that are doing surveys do get an email or text though). When data are entered, it will show in green as "up-to-date". However, you can enter the data at any time. We highly recommend you enter the data right at the end of the program, so you have the greatest potential of capturing the gains patients have made due to their participation in your program (e.g., if they stop exercising post-program and you assess 2 weeks later, their values will be poorer).

These variables are all found on page 5 of the registry. This section should be completed for ALL patients who enrolled, regardless of whether they did not complete the program.

6. **Supervised Exercise Sessions Completed**
   Enter the total number of supervised (on-site, but could be remote if the full session is supervised in real-time remotely) exercise classes completed by the patient during their rehab program (we have the number prescribed from your completed program survey; this can be used to assess program adherence). Do not count days the patient exercises independently at home as that is captured elsewhere. Alternatively, click the box if the patient is in an unsupervised program.
   Even if the patient dropped out (which will be captured in the next variable), please report # attended.

   Data Entry: click one of the 2 buttons
   If you select "supervised exercise", then a box will appear to enter the # of sessions
   Or select "patient enrolled in solely home-based program, where exercise classes are not remotely monitored in real-time"… but technology may be used (this is captured also in patient-reported items); this is basically "not applicable"

7. **Premature Program Termination / Program Completion**

   Premature termination refers to the instance where patients do not complete their prescribed exercise sessions or other core components of the program. To complete the CR program a patient must have attended at least some of the CR intervention components AND also have completed a formal re-assessment by the CR team at the conclusion of the CR intervention.

   Indicate the reason for premature termination of the patient's cardiac rehab program, if applicable. For example, a cardiac clinical event or procedure could be having bypass

Copyright © International Council of Cardiovascular Prevention and Rehabilitation
To be used with permission only. Not for re-distribution

ICCPR Intellectual Property

surgery or experiencing heart failure decompensation or exacerbation so having to stop coming. A non-cardiac clinical event or procedure could be contracting an infectious condition or cancer for example.

Data Entry: click one of the 2 buttons; more options appear if you click the first:

- ☐ Premature program termination (i.e., patient did not complete post-program assessment), for the following reason (select 1):
  - ☐ Lost to follow-up or unknown / Patient dropout for non-clinical reasons
  - ☐ Return to work
  - ☐ Clinical issue – Cardiovascular (non-fatal)
  - ☐ Clinical issue – Non cardiovascular (non-fatal)
  - ☐ Death (*note: once this is selected, this record will be denoted as complete*)
  - ☐ other
- ☐ Program completion (i.e., patient engaged in interventions and had post-program re-assessment)

ICCPR Intellectual Property

Copyright © International Council of Cardiovascular Prevention and Rehabilitation
To be used with permission only. Not for re-distribution

PROGRAM-REPORTED VARIABLES

PRE/INTAKE AND POST-PROGRAM (OBJECTIVE CLINICAL OUTCOMES)

Time windows: For each, try to get information from maximum 3 months prior to initial assessment to max two weeks after initial assessment; for post-program, the test should be done +/- 3 weeks from discharge assessment date.

Even if the patient does not complete the program, try to get the values from whatever source you can.

These variables are all found on page 2 (pre-program) and 6 (post-program) of the registry.

8. **Lipids: Low-Density Lipoprotein (LDL-C)**

   Data Source: Lab report. If you do not collect this, see if you can get the information from the primary care provider or specialist.

   Data Entry: select units (mmol/L or mg/dL). Enter value obtained for LDL to 1 decimal place

   ☐ Unknown (If there are no values within the time window above; or, the patient did not have any repeat lipid test done at discharge, for example)

9. **Body mass index**

   Enter the patient's BMI.

   Data Source: Direct measurement of height (m) and weight (kg) at the intake and discharge assessment. You can use an online calculator to compute if it is easier (e.g., https://www.nhlbi.nih.gov/health/educational/lose_wt/BMI/bmicalc.htm). If the patient does not come in for their post-program assessment, if you can ask them for their weight, you could calculate this using their height at baseline.

   Data entry: Enter value obtained from kg/m^2 to 1 decimal place if possible

10. **Systolic and diastolic blood pressure (BP; mmHg)**

Canadian Hypertension Education Program (CHEP) Recommended Technique for Measuring Blood Pressure

Place the cuff so that the lower edge is 3 cm above the elbow crease and the bladder is centered over the brachial artery. **The patient should be resting comfortably for 5 minutes in the seated position with back support.** The arm should be bare and supported with the antecubital fossa at heart level, as a lower position will result in an erroneously higher SBP and DBP. **There should be no talking, and patients' legs should not be crossed. At least three measurements should be taken in the same arm with the patient in the same position. The first reading should be discarded and the latter two averaged.**

CHEP Recommendations for accurate measurement of blood pressure: https://guidelines.hypertension.ca/diagnosis-assessment/measuring-blood-pressure/

CR Intellectual Property

Copyright © International Council of Cardiovascular Prevention and Rehabilitation
To be used with permission only. Not for re-distribution

Enter the patient's systolic blood pressure (mmHg) and the patient's diastolic blood pressure (mmHg). BP assessement should be undertaken manually or with a validated automated device only (e.g., see: **http://www.dabeducational.org/sphygmomanometers/recommended_cat.html**).

Data Source: Direct measurement at the assessment. Or if the patient does not come in for their post-program assessment ask if they have a reliable home BP monitor or could go to a pharmacy to get their blood pressure taken.

Data entry: Enter value obtained from patient's SBP and DBP.

11. **Peak METs**

Indicate the peak metabolic equivalents of task (METs) achieved during a functional / exercise capacity test or assessment (e.g., GXT, ISWT, 6MWT) in the space provided (we will know the type of test from your program survey responses). The METs can be estimated from standard equations using speed and grade, or can be calculated from the direct measurement of oxygen consumption using gas analysis.

The Duke Activity Status Index (DASI; self-report survey) can also be used, particularly if the patient does not come in for a post-program assessment. You can use this online survey for the items and calculuation (https://www.mdcalc.com/duke-activity-status-index-dasi; DASI is converted to METS by dividing the total score by 3.5).
Conversion table for 6MWT (issues if have to use shorter than 30 m passage) to METs: https://iacpr.net/resources/Documents/6MWT%20Distance%20Conversion%20Table%20.pdf
Conversion for watts to METs: https://exrx.net/Calculators/CycleMETs
Ideas on conversion of Incremental Shuttle Walk Test to METs (see Table 2 in particular): https://bjsm.bmj.com/content/42/1/36.long (see also: https://journals.lww.com/jcrjournal/Fulltext/2019/05000/Validity_of_the_Incremental_Shuttle_Walk_Test_to.13.aspx)

Data Source: Exercise stress test or other functional assessment.
Data Entry: Enter the numeric value of the peak METs as indicated by the exercise test report to the nearest 1/10 of a MET (i.e. 5.4 METs).

12. **Optional site variable**

Sites may capture another parameter of their choice at pre and post-program / progress (e.g., Could be blood glucose, other CPET parameters, participation in maintenance program), or make any notations here (e.g., Can denote if used different measurement pre and post). Numbers and letters can be entered.

ICCPR Intellectual Property

Copyright © International Council of Cardiovascular Prevention and Rehabilitation
To be used with permission only. Not for re-distribution

PATIENT-REPORTED OUTCOMES (if willing, and sufficient English language capacity, or staff administer)

PRE-PROGRAM/INTAKE only (send after receipt of referral and before first exercise session, after information about registry received by patient if possible):

These variables are all found on page 3 of the registry.

13. **Do you have someone in your life who you feel supports you emotionally and with your health?**
    - ☐ Definitely
    - ☐ Most of the time
    - ☐ Some of the time
    - ☐ Rarely
    - ☐ No I do not

14. **For how many years did you do formal schooling / education?** (note: the patient should count from first grade, so for example someone with university schooling would likely have a value greater than 13)

    ________ years

15. **Has a doctor ever told you that you have any of the following health conditions? (check all that apply)**
    Note: patient report can be augmented with program report (e.g., you can add any conditions of which you are aware from medical charts). If the patient does not provide this information, please provide what you do know from the medical records to which you have access. Note there is an open-ended text field if "other" is selected where you can add details as desired.
    - ☐ Stroke / transient ischemic attack
    - ☐ Peripheral vascular disease / claudication
    - ☐ Diabetes
    - ☐ Liver disease
    - ☐ Kidney disease
    - ☐ Lung disease (e.g., COPD, Asthma)
    - ☐ Osteoporosis
    - ☐ Cancer
    - ☐ Human immunodeficiency virus / AIDS
    - ☐ Movement disorder (e.g., parkinson's, tremor)
    - ☐ Musculoskeletal issues (e.g., arthritis, hip or knee replacement)
    - ☐ Cognitive issues (e.g., brain injury, cognitive impairment)
    - ☐ Mental health problems, including sleep issues (e.g., depression, anxiety)
    - ☐ Sexual issues (e.g., erectile dysfunction)
    - ☐ Other (please specify: ________________________________)
    - ☐ None of the listed options

ICCPR Intellectual Property

Copyright © International Council of Cardiovascular Prevention and Rehabilitation
To be used with permission only. Not for re-distribution

16. **How much do you worry about having enough money to meet your basic needs, including health and health care?**
    - ☐ Not at all
    - ☐ I sometimes worry about this
    - ☐ I often worry about this

17. **Do you have to pay for heart pills or medicines out of your own pocket?**
    - ☐ Yes, I have to pay for any medicine I take out of my own pocket, or some of the cost
    - ☐ No, I have work benefits, or the government or some other source pays for <u>all</u> my heart medicine

ICCPR Intellectual Property

Copyright © International Council of Cardiovascular Prevention and Rehabilitation
To be used with permission only. Not for re-distribution

PRE/INTAKE (if patients are not receiving these questions before they start the program, ask them to answer based on what they were doing / how they felt before their first visit), POST-PROGRAM (administered when program would have been done for all patients [which each program provided in the initial program survey], regardless of whether or not the patient completed the program; if program has no end date, send 4 months from pre-program assessment), and EACH YEAR FROM INITIAL PRE-ASSESSMENT:

These variables are all found on page 4 (pre-program), 7 (post-program/progress), and 10 (first annual) of the registry.
Subsequent annual follow-ups are generated automatically by the registry. They will appear on the main patient search / "my patients" page in the follow-up column as "assessment due" at the applicable number of years from the initial assessment date.

18. Quality of Life / Subjective Well-Being

"Assume that this ladder is a way of picturing your life. The top of the ladder represents the best possible life for you. The bottom step of the ladder represents the worst possible life for you. Circle the number that shows where on the ladder you feel you are right now."

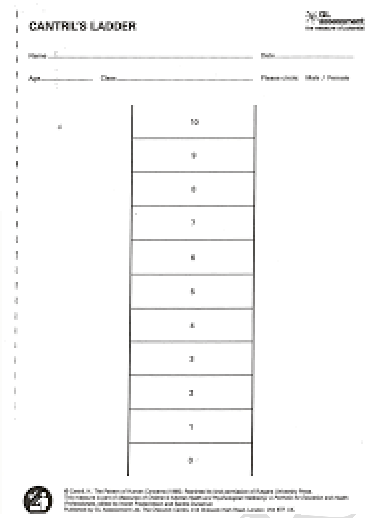

Validated measure: Cantril's ladder of life. This is also used in the Gallup World Poll, World Happiness Report etc, so is valid across many countries.
Source: Cantril H. The pattern of human concerns. New Brunswick, NJ: Rutgers University Press, 1965.
Data Entry: value between 0 (worst possible life) and 10 (best possible life)

19. Depressive symptoms (validated measure: PHQ-2):

Information about the PHQ-2 can be found here: https://www.phqscreeners.com/ (many validated translations available) and here https://en.wikipedia.org/wiki/Patient_Health_Questionnaire. This measure is in the public domain, so permission is not required to use it. Note this short 2-item version has good diagnostic sensitivity, but poor specificity. If a or b is rated "more than half the days" or "nearly every day" it is recommended the patient undergoes a diagnostic interview with a registered mental healthcare professional.

Copyright © International Council of Cardiovascular Prevention and Rehabilitation
To be used with permission only. Not for re-distribution

Over the past 2 weeks, how often have you been bothered by any of the following problems?

a) **Little interest or pleasure in doing things**
b) **Feeling down, depressed or hopeless**

Response options are: not at all (0), several days (1), more than half the days (2), nearly every day (3).

20. **In the last month, how many servings of fruit and vegetables did you have in an <u>average</u> day?** (e.g., here are some examples of one serving: 125 mL [½ cup] fresh, frozen or canned vegetables or fruit; 250 mL [1 cup] leafy green vegetables such as lettuce, which is about the size of your fist; 1 small piece of fruit or vegetable such as an apple, guava, nectarine, orange, peach, pear, large carrot or celery stalk; 60 mL [1/4 cup] of dried fruit such as raisins; or small 125mL glass of pure fruit juice [sugary drinks with fruity taste should not be counted]).

___________ servings /day

21. **In the last month, how many minutes per week on average were you physically active to the point of being at least slightly short of breath?**

__________ minutes / week

22. **Do you use any form of tobacco? (e.g., smoking, vaping etc; select 1)**

☐ Never = no history of using any form of tobacco
☐ Current = use of any form of tobacco within the last month
☐ Former = use of any form of tobacco more than one month ago

23. **It is hard to remember to take your pills all the time if you take them. Over the last month, how often do you think you have taken your heart pills as directed by your doctor? (select 1)**

☐ All the time
☐ Most of the time
☐ Some of the time
☐ Rarely
☐ Never

Note: this is scored as 1 "never" to 5 "all the time". The mean is shown in the dashboards (see separate file on reviewing reporting dashboards)

24. **What is your current work status? (select 1)**

☐ I work full or part-time for pay (includes self-employment)
☐ I am on disability (sick leave) or modified duties at work
☐ I am retired
☐ I have not been employed, or have been working without formal pay (e.g., household management)
☐ Other (e.g., can't work due to health)

ICCPR Intellectual Property

Copyright © International Council of Cardiovascular Prevention and Rehabilitation
To be used with permission only. Not for re-distribution

POST-PROGRAM ONLY (PROGRESS):

These variables are all found on page 8 of the registry.
Again, these should be administered even if the patient did not complete the program if possible.

25. **Please check whether you feel you are fully informed about each of the following ways to control your heart disease (check yes or no for each):**
    - ☐ I know that my primary care or other healthcare provider was informed about what happened for me in cardiac rehab or I informed him/her (or I do not have a primary healthcare provider)
    - ☐ I understand what heart pills I should be taking
    - ☐ I know how to manage my stress
    - ☐ I know how much I should be exercising and at what intensity, so I can stay active
    - ☐ I know how to follow a heart-healthy diet
    - ☐ I know my blood pressure level and how to control it
    - ☐ I know my cholesterol level and how to control it
    - ☐ I know what to do if I have chest pain
    - ☐ I have been supported to get back to the life roles that are important to me (e.g., relationships, domestic duties, driving)

26. Did you do any part of your cardiac rehab **online or via phone**? (does not include scheduling sessions or appointments)
    - ☐ Yes
    - ☐ No

ICCPR Intellectual Property

Copyright © International Council of Cardiovascular Prevention and Rehabilitation
To be used with permission only. Not for re-distribution

Each Year Post-Initial Assessment Date

Annual follow-ups consist of 1 program-reported variable (vital status; p. 9 of registry) and several patient-reported variables if they are willing (or the program may enter the information directly on behalf of the patient, depending on your arrangement; pages 10 and 11 of registry). On the patient search / "my patients" listing page, the "annual follow-up status" column will show as "assessment due" when it is the time to complete this assessment; there is no active alert to you to go in and enter that, so if you are worried about forgetting you can put a reminder in your electronic calendar (patients that are doing surveys do get an email or text though).

If you simply want to stop annual follow-ups by patients doing surveys due to patient request, please remove their contact information from page 1 in the registry; we would appreciate if you could still collect vital status annually until the patient has died and record it in the registry; The date of next annual assessment shall appear when due on the "my patients" screen.

Subsequent annual follow-ups are generated automatically by the registry. They will appear on the main patient search / "my patients" page in the follow-up column as "assessment due" at the applicable number of years from the initial assessment date; there is no active alert to you to go in and enter that so if you would like one, enter it in your electronic calendar (patients that are doing surveys do get an email or text though). Note: if you create a subsequent annual follow-up early to denote patient health status, if patients are completing surveys, they will not be sent when you create the annual follow-up, but when a year has passed since initial assessment. But as above, once you denote they have passed away or are too ill, the annual patient survey will not ever be sent.

Program-Reported

The patient row will show in the colour yellow on the "my patients/ search" page when this is due. Please try to report this within +/- 3 weeks of the anniversary of the initial assessment date.
This program-reported variable for the first year is found on page 9 of the registry. For subsequent years, this will be found on main "my patients" page, under the column at the far right "annual follow-ups from 2nd year".

27. Vital Status

If they agree, you ask them questions 18-24, 28 and 29 and enter in the registry. These variables are found on page 11 of the registry (for the first year); subsequent years will appear on the "patient search/ my patients" page when they are due under "annual follow-ups from 2nd year".

Please note that in the registry, on the main page you can see when the next annual follow-up is due for each patient on the right.

- ☐ patient died (*note: once this is selected, this record will be denoted as complete*)
- ☐ patient alive
- ☐ patient too ill to complete any further assessments (e.g., moved to nursing facility, dementia, palliative, disabling stroke; *note selecting this will cease further emails/texts to patients reporting data*)
- ☐ Could or did not confirm

ICCPR Intellectual Property

Copyright © International Council of Cardiovascular Prevention and Rehabilitation
To be used with permission only. Not for re-distribution

Patient-Reported

These variables are found on page 11 of the registry (for the first year); subsequent years will appear on the “patient search/ my patients” page when they are due under “annual follow-ups from 2nd year”. See above how to cease follow-ups due to patient death, illness or request.

28. How often have you felt short of breath, dizziness or had chest pain on average in the past month?
    - ❑ Never
    - ❑ Rarely
    - ❑ Sometimes
    - ❑ Often
    - ❑ Always

29. Have you been hospitalized or had another heart or other health problem in the past year?
    - ☐ *Yes*
    - ☐ No

    29b. If yes, was it (check yes or no for each):
    - ☐ A heart attack
    - ☐ A stroke or mini-stroke (“brain attack”; transient ischemic attack)
    - ☐ I went to the emergency department for a heart problem (e.g., chest pain, heart failure)
    - ☐ I stayed in the hospital overnight for a heart problem
    - ☐ I had a heart procedure (e.g., bypass surgery, stent, rhythm device inserted like a pacemaker)
    - ☐ I went to the hospital for a reason other than my heart
    - ☐ I had another new health diagnosis (e.g., kidney problems, diabetes, cancer, cognitive impairment) by my doctor
    - ☐ I think I am sick, but have not seen a doctor
    - ☐ Other

ICCPR Intellectual Property

Copyright © International Council of Cardiovascular Prevention and Rehabilitation
To be used with permission only. Not for re-distribution
